# Single-strand DNA-binding protein suppresses illegitimate recombination in *Escherichia coli*, acting in synergy with RecQ helicase

**DOI:** 10.1038/s41598-024-70817-5

**Published:** 2024-09-03

**Authors:** Isidoro Feliciello, Sven Ljubić, Edyta Đermić, Siniša Ivanković, Davor Zahradka, Damir Đermić

**Affiliations:** 1 Department of Clinical Medicine and Surgery, University of Naples Federico II, Napoli, Italy; 2 Division of Molecular Biology, Ruđer Bošković Institute, Bijenička 54, 10 000 Zagreb, Croatia; 3 Division of Phytomedicine, Department of Plant Pathology, University of Zagreb Faculty of Agriculture, Zagreb, Croatia; 4 Division of Molecular Medicine, Ruđer Bošković Institute, Zagreb, Croatia

**Keywords:** SSB protein, Genome stability, Truncated SSB, SSB overproduction, λ Spi^−^ assay, Molecular biology, Bacterial genetics, Genetics, Gene expression, Genomic instability

## Abstract

Single-strand DNA-binding proteins SSB/RPA are ubiquitous and essential proteins that bind ssDNA in bacteria/eukaryotes and coordinate DNA metabolic processes such as replication, repair, and recombination. SSB protects ssDNA from degradation by nucleases, while also facilitating/regulating the activity of multiple partner proteins involved in DNA processes. Using Spi^−^ assay, which detects aberrantly excised λ prophage from the *E. coli* chromosome as a measure of illegitimate recombination (IR) occurrence, we have shown that SSB inhibits IR in several DSB resection pathways. The conditional *ssb-1* mutation produced a higher IR increase at the nonpermissive temperature than the *recQ* inactivation. A double *ssb-1 recQ* mutant had an even higher level of IR, while showing reduced homologous recombination (HR). Remarkably, the *ssb* gene overexpression complemented *recQ* deficiency in suppressing IR, indicating that the SSB function is epistatic to RecQ. Overproduced truncated SSBΔC8 protein, which binds to ssDNA, but does not interact with partner proteins, only partially complemented *recQ* and *ssb*-*1* mutations, while causing an IR increase in otherwise wild-type bacteria, suggesting that ssDNA binding of SSB is required but not sufficient for effective IR inhibition, which rather entails interaction with RecQ and likely some other protein(s). Our results depict SSB as the main genome caretaker in *E. coli*, which facilitates HR while inhibiting IR. In enabling high-fidelity DSB repair under physiological conditions SSB is assisted by RecQ helicase, whose activity it controls. Conversely, an excess of SSB renders RecQ redundant for IR suppression.

## Introduction

Genome stability is of paramount importance to all living organisms. Genome instability, caused by aberrant DNA rearrangements (e.g., deletions, amplifications, translocations, etc.), gives rise to severe conditions such as low viability in bacteria and eukaryotes as well as cancer, sterility and premature aging in vertebrates. The RecQ family of evolutionarily conserved proteins is considered the main genome caretaker in bacteria and eukaryotes, whose members both initiate the homologous recombination (HR) DNA repair pathway and disrupt aberrant DNA structures with their 3ʹ–5ʹ helicase activity^1–3^. Stability of the *Escherichia coli* genome is determined by the metabolism of 3ʹ-ending single strand tails at DNA double strand breaks (DSBs), which are faithfully mended by HR catalyzed by RecBCD enzyme in wild type (wt) cells^4–7^. Interestingly, the efficient DSB repair by RecBCD renders RecQ's role minor in *E. coli* genome preservation^4^. The importance of HR for DSB repair in *E. coli* is manifested by its robustness. Namely, HR occurs even when RecBCD is either mutated or completely absent from a cell, which is how different HR pathways are defined in the bacterium, as reviewed^7^. DSB repair by HR is quite effective in *recD* and *recB1080* mutants, wherein changes in RecBCD composition and function include loss of its RecD subunit or inactivation of its lone nuclease domain, respectively (reviewed in^7^). Mutants lacking all RecBCD functions are also proficient in DSB repair when they are deficient in ExoI and SbcCD exonucleases, and the functions of RecBCD are complemented by RecQ and UvrD helicases, RecJ exonuclease and RecFOR recombination mediating proteins (reviewed in^7^).

**Fig. 1:**
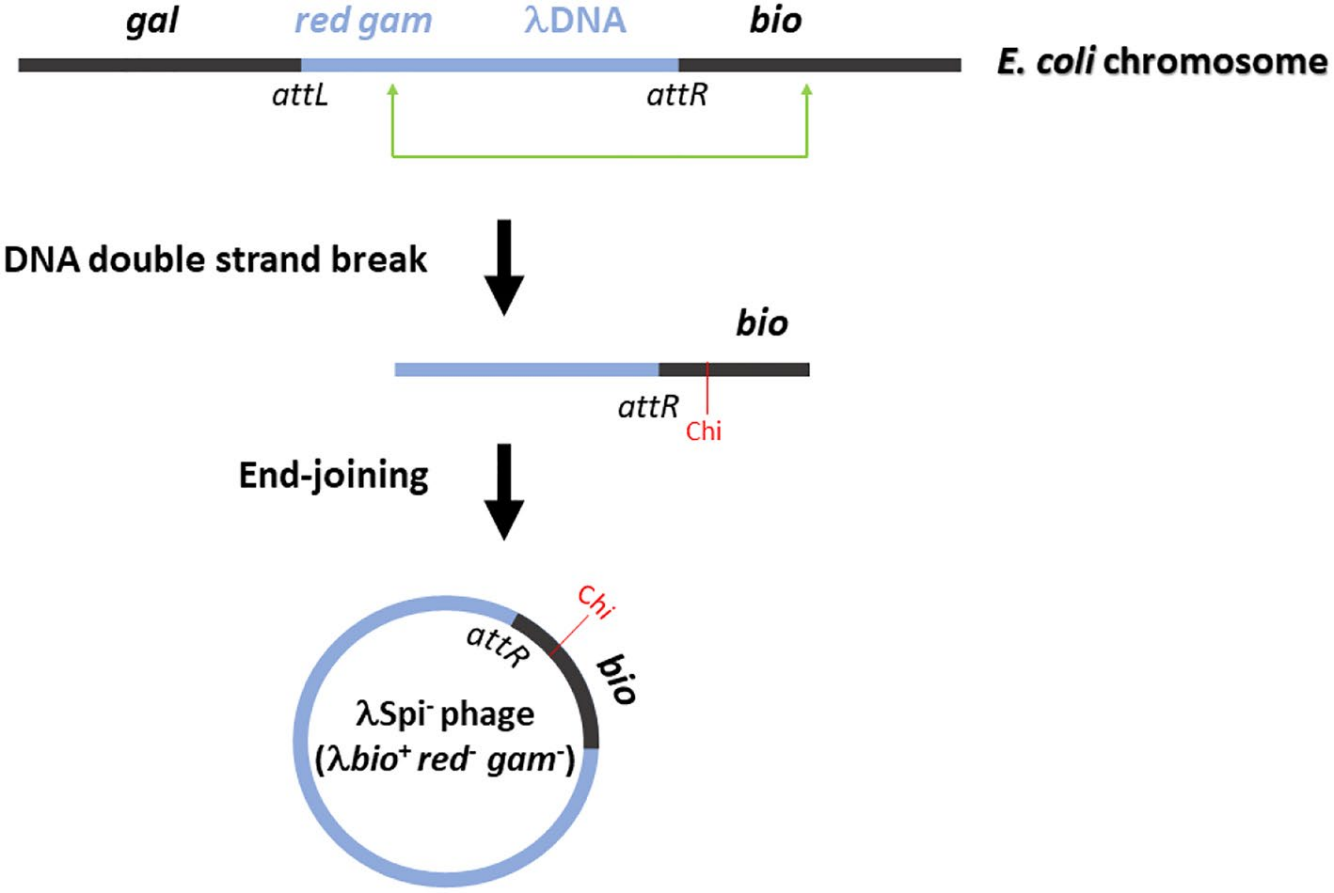
In vivo assay for illegitimate recombination in *E. coli* (modified from^10^). Due to aberrant recombination events between the λ phage and neighboring *E. coli* genomic DNA during the excision of the prophage, a transducing λ *bio* phage is produced, which lacks the *red* and *gam* phage genes. These phages can be detected by an *E. coli* P2 lysogen strain, where they produce large infectious centers (full Spi^−^ phenotype) since their DNA is protected from degradation by the RecBCD enzyme through a Chi sequence in the *bio* gene^11^. The wild type λ phage does not contain Chi.

However, occasionally aberrant DNA transactions occur in *E. coli* genome resulting in illegitimate recombination (IR) events. IR is mostly suppressed by the RecQ helicase, as reported in a seminal paper by Ikeda’s group^1^, which was the first to characterize RecQ as a genome caretaker (using λ Spi^−^ assay). The increased level of IR in *E. coli* is correlated with decreased cellular viability and reduced HR^4,5^. The λ Spi^−^ assay is effectively used to quantify the frequency of IR in *E. coli* genome^8^. It detects an aberrantly excised λ prophage that contains a part of bacterial genome (*bio* gene containing a Chi site) instead of its own *red* and *gam* genes (Fig. 1), and such a phage produces  a large infective center on P2 lysogenic bacteria (the Spi^−^ phenotype^9^), unlike wt λ phages. Some of the distinguishing features of the *E. coli* IR detected by the λ Spi^−^ assay include their origin from disturbed DNA replication and ensuing DSBs resection^10^, with the thus-produced 3ʹ overhangs aligning broken DNA ends by an end-joining reaction that is independent of RecA recombinase, but does rely on microhomologies (of around 9 bp) and on ligase activity^9,10^. There is a balance of IR and HR occurrence in *E. coli*, which is determined by the DSB resection^4^.

Bacterial SSB proteins, as well as their eukaryotic RPA analogues, are essential and ubiquitous. They avidly bind single-stranded DNA (ssDNA) and regulate/coordinate its metabolism, hence enabling essential DNA processes such as replication, HR and repair. There are two mechanisms of SSB action in a cell: SSB binds to ssDNA in a sequence-independent manner and protects it from the activity of various nucleases while concomitantly the reducing reactivity of the ssDNA by sequestering it^12,13^. Moreover, SSB interacts with/recruits multiple enzymes involved in DNA metabolism, thus acting as a molecular matchmaker for at least 20 proteins that comprise the SSB interactome in *E. coli*^14–16^. Notably, in addition to single-strand dependent exonucleases, some helicases and polymerases, SSB recruits RecQ helicase to ssDNA and stimulates its helicase activity^17,18^. Out of 178 amino acids that constitute *E. coli* SSB protein, it is the conserved C-terminal amphipathic tip of 8 amino acids that mediates interactions with other proteins, whereas the conserved N-terminal domain (of 115 amino acids) is required for homotetramer formation and cooperative binding to ssDNA^15,19–21^.

Two previous reports indicate that SSB influences genome stability in *E. coli* as evidenced by frequency of precise transposon excision^22^ or by the level of deletions formed in it^23^.

Moreover, it was reported earlier that RPA, a eukaryotic SSB analog, prevents annealing between short-sequence homologies and thus suppresses microhomology-mediated end joining (MMEJ) repair of DSBs, while promoting the HR pathway^24,25^. We noticed earlier that MMEJ shares multiple analogies with *E. coli* IR^4^ and therefore here we assessed the effect of SSB protein on IR occurrence in *E. coli* genome.

## Results

We used the well-established λ Spi^−^ assay to detect IR in *E. coli*^8,10^. Since the *ssb* gene is essential, we could not inactivate it completely and therefore we used conditional, thermosensitive mutation *ssb-1* to temporarily and reversibly inactivate SSB by shifting bacteria to 42 °C, which also served to thermo-induce prophage λ*cI857* excision from the bacterial chromosome and start its lytic cycle.

### SSB protein suppresses IR in *E. coli*

**Fig. 2:**
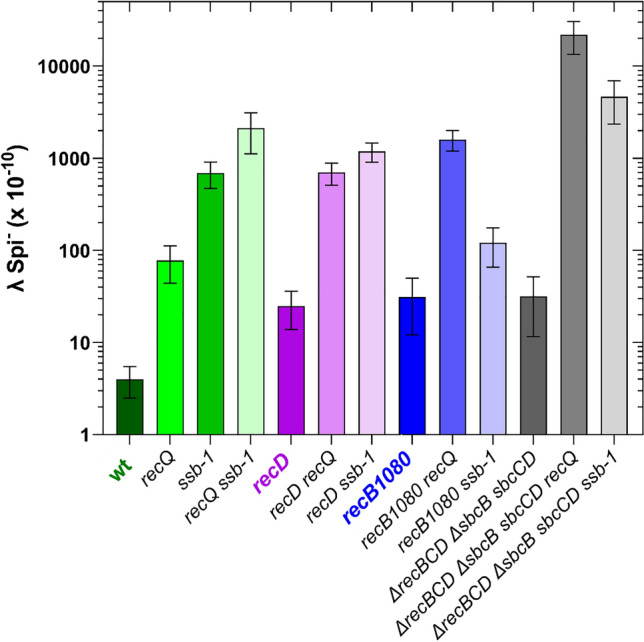
Illegitimate recombination in wt, *recD*, *recB1080* and *recBCD sbcB sbcCD* genetic backgrounds is inhibited by SSB protein. Incubation at 42 °C temporarily/reversibly inactivates SSB-1 protein as well as inducing lytic cycle of λ*cI857* prophage. The data represent the mean of at least three independent experiments ± standard deviation.

The rate of IR observed in wt bacteria was about 4 × 10^−10^ (Fig. 2), which is comparable to our earlier results^4,5^. As expected and previously observed^4^, inactivation of the RecQ led to approximately 20-fold increase of IR (Fig. 2). Importantly, thermal inactivation of SSB-1 protein increased the frequency of IR by more than 170-fold compared to the wt strain DE105 (Fig. 2). Since RecQ is considered to be the strongest IR suppressor in *E. coli*, and its activity is directed and assisted by the SSB protein, we monitored IR in a strain (DE743) where both RecQ and SSB were inactive, and observed about 27-fold increase in IR compared to a single *recQ* mutant DE111, while the increase was over 530-fold compared to wt strain (Fig. 2). Notably, the double *recQ ssb-1* mutant had about triple the IR frequency of the *ssb-1* mutant (Fig. 2), and this difference is significant (P = 0.0029, n = 8, two-tailed *t*-test).

In a *recD* mutant background, inactivation of either RecQ or SSB caused about 28-fold and 47-fold IR increase, respectively (Fig. 2). This suggests that both RecQ and SSB suppress IR in bacteria that exhibit only the helicase and RecA loading activity of a RecBC enzyme, while lacking the nuclease and Chi recognition activity of the RecBCD holoenzyme.

Similarly, in the *recB1080* mutant background, both RecQ and SSB inactivation produced an increase in IR. However, unlike the previous cases, the effect of RecQ inactivation was stronger (approximately 51-fold) than that of SSB (about fourfold) (Fig. 2), compared to the parental *recB1080* mutant strain DE153 (whose IR rate is higher than that of the wt, as observed earlier^4,5^).

Finally, bacteria devoid of all RecBCD functions, but containing suppressor mutations that enable DSB repair (strain DE762), showed about an eightfold increase in IR compared to the wt strain even with active RecQ and SSB (Fig. 2). IR frequency strongly increased upon inactivation of either RecQ (about 700-fold) or SSB (about 150-fold) (Fig. 2), suggesting that in this genetic background both proteins suppress IR.

Based on the overall results, we conclude that both RecQ and SSB suppress IR across all recombination pathways for DSB repair in *E. coli*, indicating that this inhibition is general characteristic in *E. coli*. The additive effects of their inactivation suggest that RecQ and SSB act at different steps of IR inhibition. The baseline level of IR was lowest in wt bacteria, compared to mutants with active alternative pathways for DSB resection, indicating the adaptation of wt bacteria to preserving genome stability.

#### Residual activity of SSB-1 protein at nonpermissive temperature

Since *ssb-1* is not a null mutation, but rather a conditional (thermosensitive) one, we checked the residual activity of the SSB-1 protein at the nonpermissive temperature of 42 °C. The activity of SSB-1 is increased by rising NaCl concentrations^26^, so we tested the level of IR in bacteria grown in LB medium containing either 10 g/l or 2 g/l of NaCl.

**Fig. 3:**
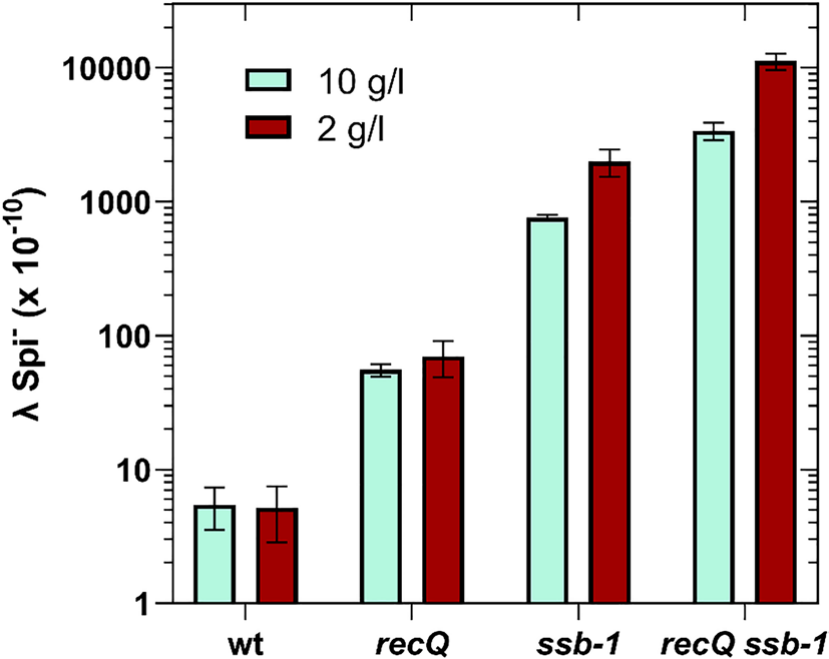
Illegitimate recombination in the *ssb-1* and *recQ ssb-1* mutants is more pronounced in LB medium containing lower NaCl concentration. Each value is an average of three independent experiments, with error bars representing standard deviation.

As shown in Fig. 3, wt and *recQ* mutant strains showed no significant difference in IR levels with respect to their growth in the two media (P > 0.4469, two-tailed *t*-test). On the other hand, *ssb-1* and *ssb-1 recQ* mutants grown in medium depleted of NaCl had 2.67 and 3.15-fold higher IR, respectively, than when grown in medium enriched with NaCl, which is significant (P = 0.0148 and 0.0032, respectively, two-tailed t-test).

Therefore, we conclude that the SSB-1 protein is not completely inactive at the nonpermissive temperature in our experimental conditions (10 g/l NaCl, as a lower concentration in the medium reduces λ phage burst size). This indicates that the role of SSB in preventing IR may be underestimated in our study.

### SSB and RecQ are required for efficient HR

**Fig. 4:**
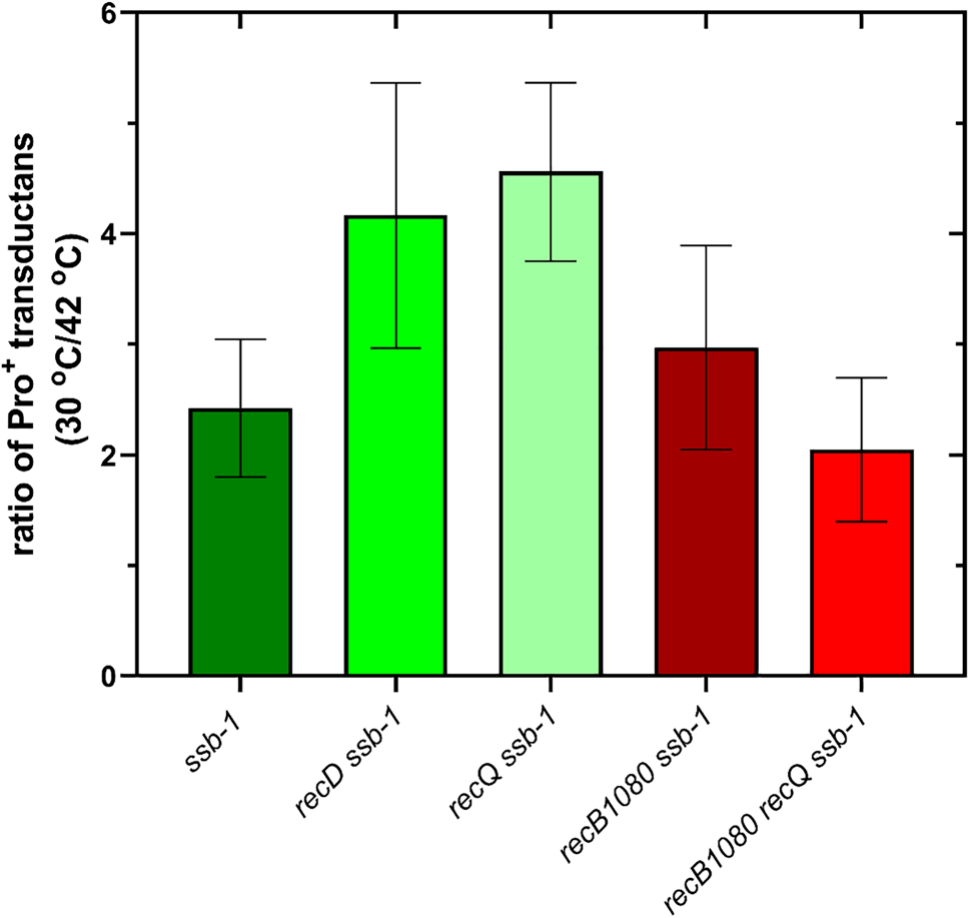
Homologous recombination in *ssb-1* derivatives of wt, *recD* and *recB1080* strains is impaired at the nonpermissive temperature in P1 transduction crosses. Each value is an average of three independent experiments, with error bars representing standard deviation.

It was previously reported that the *ssb-1* mutation reduces the HR rate by about five-fold in *E. coli*^27^. We determined the efficiency of HR by P1 transduction in *ssb-1* mutants at  both the permissive and nonpermissive temperatures. In our assay, HR was reduced by about 2.5-folds in an otherwise wt strain (Fig. 4). In a RecQ deficient strain, SSB-1 inactivation led to an even stronger reduction in HR, about 4.5-fold (Fig. 4) indicating a higher requirement for SSB function in the absence of RecQ. The effect of their inactivation was again additive, as it was for IR. In the *recD* genetic background, SSB-1 inactivation caused stronger HR rate reduction (about fourfold) than in the wt strain (Fig. 4). In the *recB1080* mutant RIK174, SSB-1 inactivation resulted in about threefold reduction in HR, which was not further significantly decreased upon RecQ inactivation (Fig. 4) (P = 0.2301, two-tailed *t*-test).

We thus infer that SSB function is required for efficient HR in *E. coli* with intact RecBCD function, as well as in mutants with partially impaired RecBCD function.

### SSB protein overproduction complements RecQ deficiency

Next, we characterized the effect of SSB protein overproduction on the occurrence of IR in *E. coli*. It is known that SSB overproduction partially impairs DNA repair in *E. coli*^28–30^, but it can also enhance DNA photorepair^31^.

**Fig. 5:**
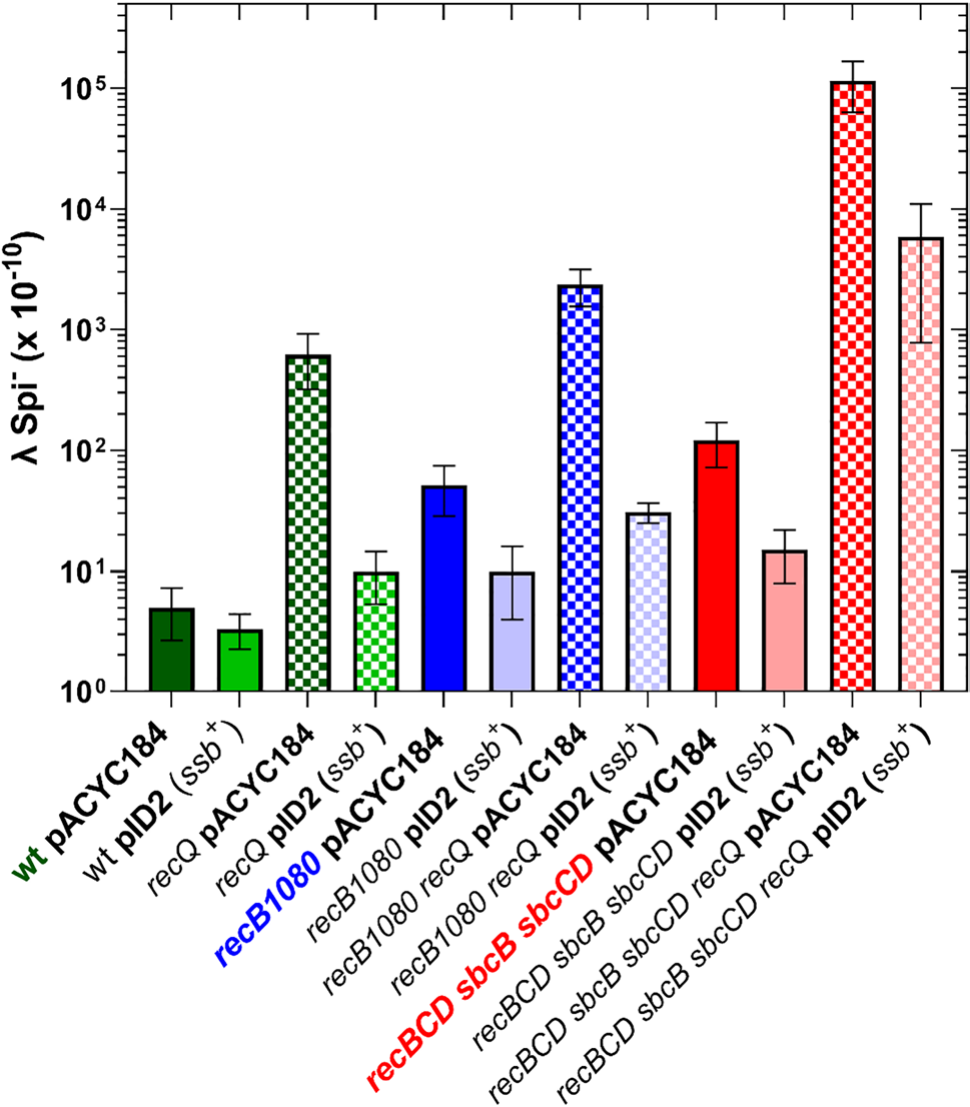
*ssb* overexpression reduces illegitimate recombination in both RecQ^+^ and RecQ deficient bacteria in wt, *recB1080* and Δ*recBCD* Δ*sbcB sbcCD* genetic backgrounds. Each value is an average of three independent experiments, with error bars representing standard deviation.

We used our recently-designed SSB overproduction plasmid pID2^30,32^, which consists of *ssb* gene cloned into low copy-number plasmid along with its natural promoters. Wild-type bacteria carrying pID2 showed slightly reduced IR, which was not significantly different from the wt strain with normal *ssb* gene expression (Fig. 5) (P = 0.3225, two-tailed *t*-test). On the other hand, SSB overproduction reduced IR in the *recQ* mutant by about 50-fold (Fig. 5), showing that an excess of SSB may compensate for the RecQ deficiency. The same effect was observed in the *recB1080* mutant, where an excess of SSB reduced IR frequency (Fig. 5). Similarly, the highly elevated IR in RecQ-deficient derivatives of *recB1080* (DE154) and Δ*recBCD* Δ*sbcB sbcCD* (DE785) was greatly reduced by overproduced SSB (Fig. 5).

Therefore, we conclude that SSB overproduction decreases the frequency of IR, as opposed to SSB inactivation. RecQ-deficient mutants also showed reduced IR upon SSB overproduction, further confirming their independent yet overlapping roles in the cell.

### SSB protein lacking its C-terminal acidic tip only partially complements RecQ and SSB deficiency

The 8 conserved C-terminal amphipathic amino acids are responsible for SSB interactions with its partner proteins, but not for SSB DNA binding^33^. We deleted this region to differentiate the role of SSB's DNA binding function in IR suppression from its protein interaction function (which includes RecQ helicase, among other proteins). For that purpose, we constructed several plasmids that carry either completely functional *ssb* gene (pSID4, analogous to pID2), or its derivative lacking promoters (pSID1, negative control) or the C-terminal tip (pSID3).

**Fig. 6:**
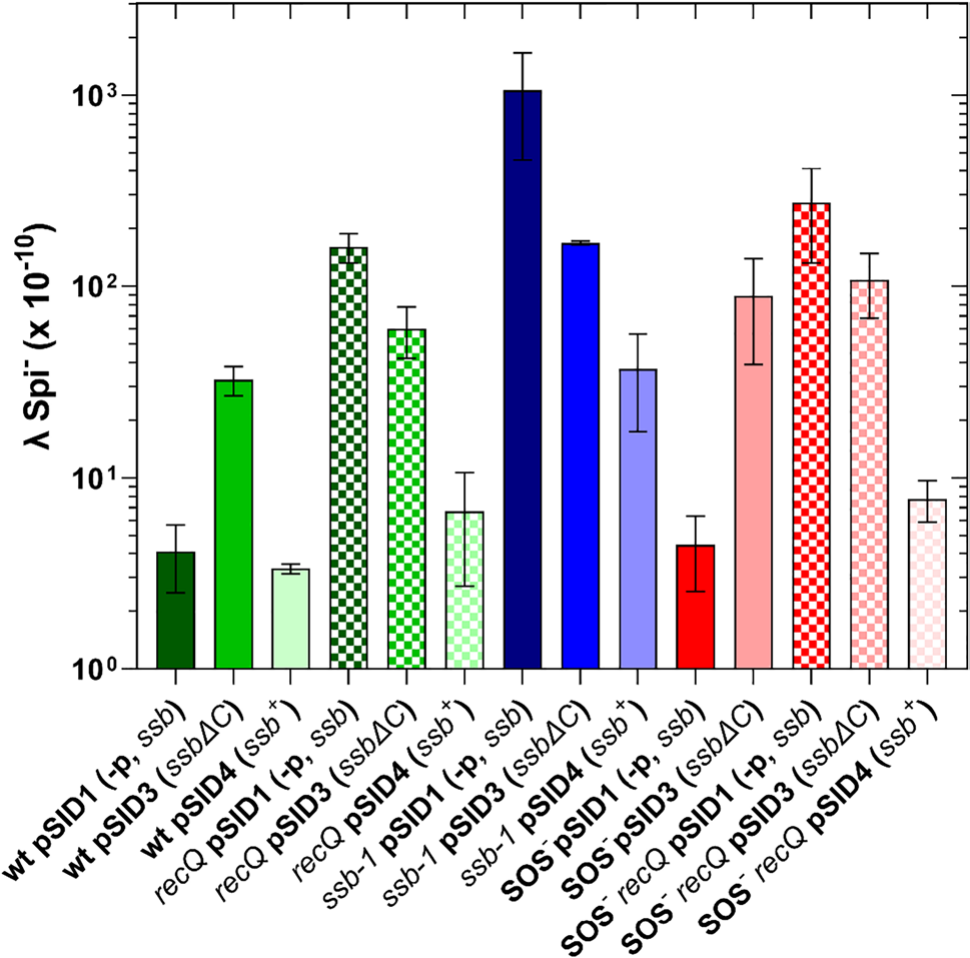
Truncated SSB protein, lacking 8 amino acids C-terminal tip, only partially complements RecQ and SSB deficiencies in inhibiting illegitimate recombination in wt and SOS-deficient genetic backgrounds. The plasmid genotype designations *ssb*^+^; -p, *ssb*; and *ssb*∆C represent the following: promoters with the whole coding region, coding region without promoters, and promoters with  a truncated coding region (lacking 8 amino acids), respectively. Each value is an average of three independent experiments, with error bars representing standard deviation.

As shown in Fig. 6, the plasmid pSID1, which carries the inactive *ssb* gene, did not interfere with IR in the wt strain, and its rate was about 4 × 10^−10^. Overproduction of wt SSB from the plasmid pSID4 reduced IR frequency, but the difference was not significant (P = 0.4279, two-tailed *t*-test), unlike the overproduction of a truncated SSBΔC8 protein, which caused about an eightfold increase in IR (Fig. 6) (P = 0.0011, two-tailed *t*-test). Furthermore, an excess of the truncated SSBΔC8 protein moderately (but significantly, P = 0.0035, two-tailed *t*-test) reduced the IR in the *recQ* mutant (Fig. 6), whereas overproduction of wt SSB decreased the IR to almost the wt level (Fig. 6), which is consistent with the effect of the pID2 on the *recQ* mutant (Fig. 5).

Overproduction of the truncated SSBΔC8 protein partially complemented the inactive SSB-1 protein, leading to about a 6.5-fold reduction in IR (Fig. 6). However, IR the reduction in IR was more pronounced (about 28-fold) when wt SSB was overproduced in the *ssb-1* mutant, although its IR level remained significantly higher (about ninefold) than in the wt strain (P = 0.0435, two-tailed *t*-test, Fig. 6). This reflects the situation where a mixture of SSB-1 and an excess of SSB was present in a cell.

Since we observed induction of SOS regulon in cells producing the truncated SSBΔC8 protein (see below), we tested the effect of the SSBΔC8 protein in a mutant with an uninducible SOS system. In these cells, IR increased about 20-fold, which is more than twofold higher than in SOS proficient cells (Fig. 6). The *recQ* mutant deficient in SOS induction also showed partial complementation by the pSID3 plasmid (producing SSBΔC8 protein), while its pSID4 counterpart (producing wt SSB) caused a stronger IR reduction (about 35-fold) (Fig. 6).

Our results indicate that an excess of the truncated SSBΔC8 protein is unable to fully complement the missing RecQ or SSB-1 function, while wt SSB overproduction is unable to effectively complement the inactive SSB-1 protein.

### ssb and *sulA* gene expression

Since our study includes complementation and SSB overproduction tests, we measured the expression of the *ssb* gene by RT-qPCR. Moreover, we determined the expression of the *sulA gene*, which commonly serves as a measure of SOS regulon induction in a bacterial population^32,34^.

**Fig. 7:**
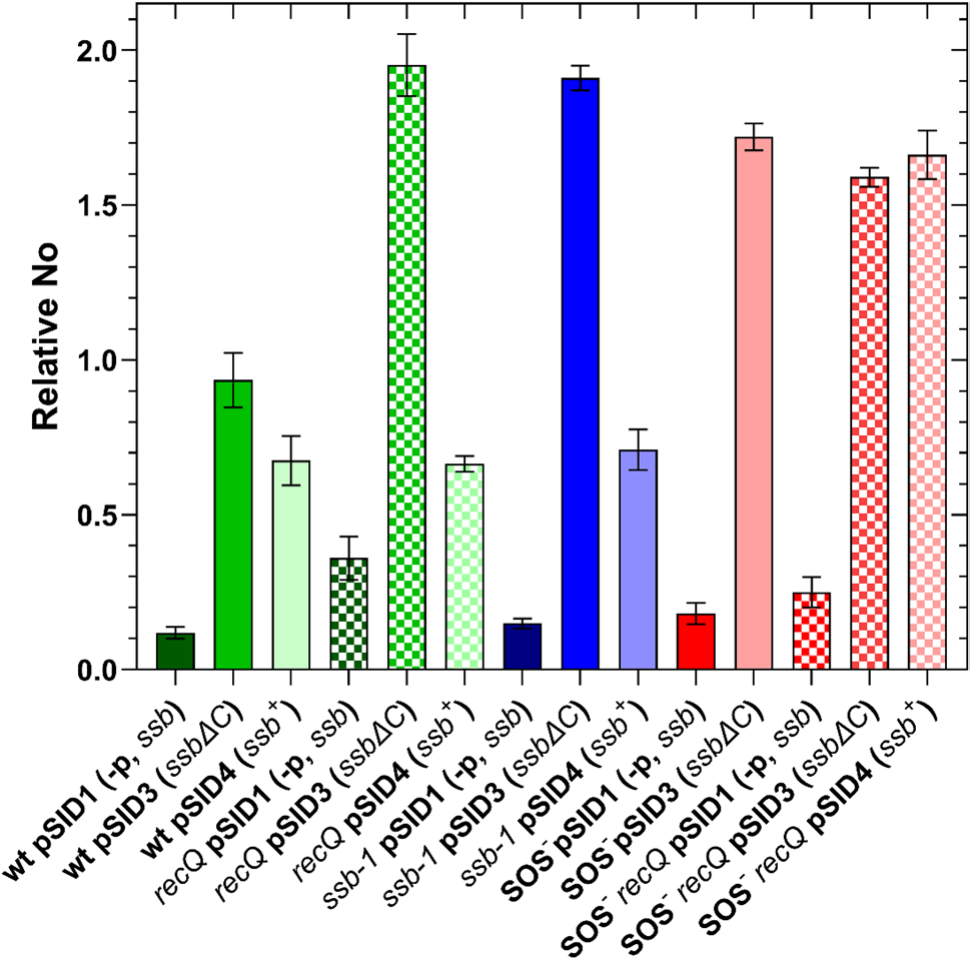
Expression of the *ssb* gene in *E. coli* carrying overexpression plasmids, grown in LB supplemented with chloramphenicol at 30 °C until reaching OD_600_ ~ 0.4. No represents the normalized No value for the *ssb* gene. Plasmid genotype designations *ssb*^+^; -p, *ssb*; and *ssb*∆C represent promoters with whole coding region, coding region without promoters, and promoters with a truncated coding region (lacking 8 amino acids), respectively. The presented data are an average of three independent RT-qPCR experiments, with error bars representing standard deviation.

As shown in Fig. 7, cultures harboring plasmids containing either the wt *ssb* gene or its truncated form showed increased gene expression in wt, *recQ* and *ssb-1* mutant strains, as well as in SOS-deficient bacteria. Expression of the *ssb* gene in bacteria carrying the plasmid pSID4 (*ssb*^+^) increased by ~ sixfold, ~ twofold and ~ fivefold compared to their respective wt, *recQ* and *ssb-1* negative controls that harbor the pSID1 plasmid (Fig. 7). Expression of the truncated *ssbΔ*C gene (from the pSID3 plasmid) was elevated by approximately eightfold, 5.5-fold, and 13-fold compared to their respective wt, *recQ* and *ssb-1* negative controls (Fig. 7), while in SOS^−^ mutant the overexpression was approximately ninefold higher (Fig. 7). In the SOS^−^ *recQ* mutant, both plasmid expressing the *ssb*^+^ gene and the one expressing the *ssbΔ*C gene elevated expression levels by approximately 6.5-fold (Fig. 7).

**Fig. 8:**
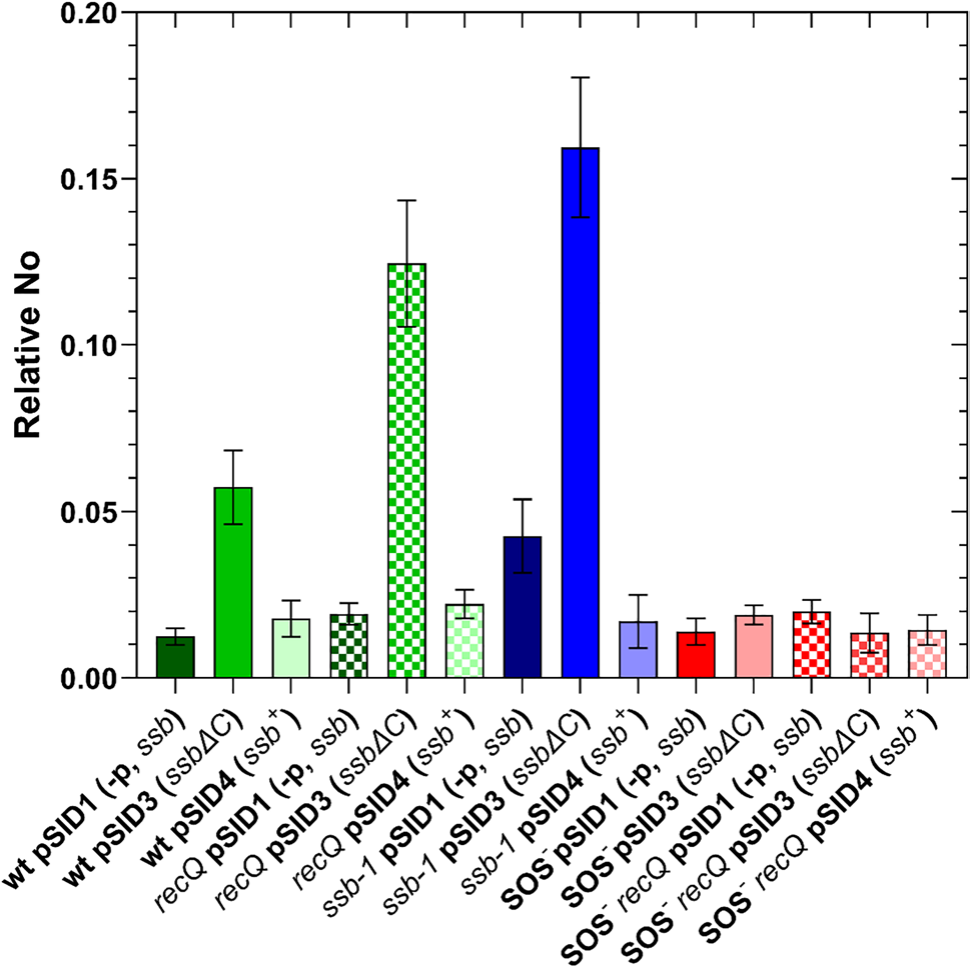
Expression of the *sulA* gene in *E. coli* carrying overexpression plasmids and grown in LB supplemented with chloramphenicol at 30 °C until reaching OD_600_ ~ 0.4. No represents the normalized No value for the *sulA* gene. Plasmid genotype designations *ssb*^+^; -p, *ssb*; and *ssb*∆C represent promoters with the whole coding region, coding region without promoters, and promoters with a truncated coding region (lacking 8 amino acids), respectively. The presented data are an average of three independent RT-qPCR experiments, with error bars representing standard deviation.

The *sulA* gene  expression in bacteria carrying the pSID1 plasmid (containing the inactive *ssb* gene, *i.e.,* negative control), was essentially equal across the wt, *recQ,* SOS^−^ and SOS^*−*^* recQ* strains*,* while it was elevated approximately  3.5-fold in the *ssb-1* mutant (Fig. 8). These results suggest that the *ssb-1* mutation causes SOS induction even when grown at the permissive temperature (30 °C). Similarly, the overexpression of wt *ssb* also did not affect *sulA* expression, except in the *ssb-1* mutant (Fig. 8), which showed ~ 2.5-fold reduction in *sulA* expression, thus indicating suppression of SOS induction in the *ssb-1* mutant by overproduced wt SSB protein. Conversely, the overexpression of the truncated *ssbΔ*C gene resulted in increased *sulA* expression in wt (~ 4.5-fold), *recQ* (~ 6.5-fold) and *ssb-1* (~ 3.5-fold) strains (Fig. 8), but not in bacteria with an inactive SOS regulon, as expected (Fig. 8). The strain containing a mixture of SSB-1 protein and an excess of truncated SSBΔC8 protein had the highest *sulA* expression, which was ~ 13-fold higher than in the wt strain, indicating strong SOS induction.

Our collective results demonstrate that overexpression plasmids increased *ssb* expression, to different extents, with the expression of the *ssbΔ*C consistently being higher (~ 2.5-fold) than that of the *ssb*^+^,  despite both sharing the same promoters. The only exception is the SOS^−^ strain, indicating that the difference in expression is due to SOS induction in cells overexpressing the *ssbΔ*C gene.

## Discussion

Using λ Spi^−^ genetic assay, we have shown that the SSB protein strongly suppresses IR while, conversely, enabling HR, thus critically supporting *E. coli* genomic stability. Since the IR detected by the  λ Spi^−^ assay originate from DSB resection^10^, we tested the role of SSB in IR suppression in several different genetic pathways of DSB resection and found that SSB suppressed IR in all cases, indicating its general character in *E. coli*.

An intriguing question concerns the relationship between SSB and RecQ functions in inhibiting IR in *E. coli*. Although the RecQ helicase family is generally considered to be the main genome caretaker in bacteria and eukaryotes^35^, several of our observations indicate that the *ssb* gene is epistatic to *recQ.* Namely, in the wt background the single *ssb-1* mutant showed a considerably higher IR than the *recQ* mutant, while the double *ssb recQ* mutant had an additionally increased IR level. Cells with inactive SSB and RecQ had a heavily increased IR (~ 530-fold) and strongly reduced HR (~ 4.5-fold), highlighting their importance for efficient DSB processing in the wt background.

Furthermore, a dominant effect on IR suppression by SSB compared to RecQ was observed in complementation tests. Namely, SSB overproduction complemented RecQ deficiency, hence indicating a role for SSB in the prevention of IR by means of a mass effect (comparing the level of IR in the *recQ* mutant with wt level of SSB and the same mutant overproducing SSB, Figs. 5 and 6), which is independent of RecQ function. We thus infer that SSB suppresses IR in two ways, depending on its concentration in a cell. When present at a physiological level, the SSB acts along with RecQ helicase, whereas an excess of SSB annuls the RecQ requirement. These observations are consistent with the role of SSB in preventing the occurrence of aberrant DNA structures that lead to IR (upstream regulation), whereas the RecQ canonically acts downstream, by disrupting such already formed structures. However, since RecQ activity on DNA is mediated by SSB^17,18^, the RecQ role in IR inhibition may as well directly depend on SSB. We addressed that possibility by using a mutant SSBΔC8 protein, lacking its conserved C-terminal tail, which binds to ssDNA but is unable to interact with its partner proteins, including RecQ^17,36^. The overexpression of the plasmid-borne *ssbΔC* gene led to an increase in IR in the wt strain (making it a partial *ssb-1* and *ΔrecQ* phenocopy), and to incomplete complementation of *recQ* and *ssb-1* phenotypes (Fig. 6). The (moderately) reduced IR in a *recQ* mutant overproducing the SSBΔC8 protein suggests that a certain aspect of SSB’s role in IR prevention is independent of its interaction with *recQ* and is likely solely due to SSB’s binding to ssDNA. This assertion is further substantiated by the ability of SSBΔC8 protein overproduction to partially complement the deficiency of the *ssb-1* mutant in suppressing IR. Namely, the SSBΔC8 protein binds ssDNA unlike the SSB-1 protein at the nonpermissive temperature. On the other hand, the ability of the overexpressed truncated SSBΔC8 protein to inhibit IR in the *recQ* deficient mutant was more limited compared to the wt SSB (Fig. 6) indicating that the interaction of SSB with some other protein(s) is relevant for IR inhibition. We thus conclude that while SSB binding to ssDNA is indeed a prerequisite for suppressing IR, it is not enough for an efficient anti-IR activity, for which interaction with RecQ (and likely some other proteins) is required.

Cells that produce truncated SSB, lacking 10 C-terminal amino acids, are not viable^37^. Here we have shown that the overproduction of SSB lacking 8 C-terminal amino acids is not lethal for the otherwise wt *E. coli*, which coproduces wt SSB from its genomic allele, nor for the *ssb-1* mutant at the permissive temperature. However, the toxicity of the SSBΔC8 protein is evident from the SOS induction in the cells producing it. Similarly, we observed the SOS induction in the *ssb-1* mutant at the permissive temperature, which readily explains the previously observed increase (2 to 3- fold) in mutagenesis of that mutant^38,39^, and is indicative of partially impaired SSB-1 protein function at the permissive temperature. Adding to that, we noted residual activity of SSB-1 at the nonpermissive temperature, which certainly understates the importance of SSB in preventing IR as well as in enabling HR (in which case it is combined with suboptimal SSB-1 function at permissive temperature). This residual SSB-1 activity may explain the increased level of IR in the *ssb-1 recQ* mutant compared to the *ssb-1* mutant, which is not expected considering the epistasis of the *ssb *gene to the *recQ* gene.

Our collective results show that SSB suppresses IR, while promoting faithful DSB repair by HR, and is therefore crucial in preserving *E. coli* genomic stability. SSB’s central role in protecting genome integrity is further aided by RecQ helicase, which itself is directed by SSB. Remarkably, the requirement for RecQ in suppressing IR was annulled by increasing the concentration of SSB, which clearly emphasizes the dominant role of SSB in preserving genome stability. Indeed, while SSB alone is sufficient for inhibiting IR (but only at an elevated concentration), these conditions are far from optimal for the cell since DNA repair itself is impaired^28–30,40^. By utilizing RecQ, *E. coli* suppresses IR at the lower SSB concentration, which does not impair other important DNA processes. Accordingly, we have recently reported that *ssb* gene expression in *E. coli* is tightly regulated by the SOS regulon and that its basal level can only be increased through heavy SOS induction^34^.

Notably, *ssb* gene transcription is coregulated with *uvrA* gene expression through a shared SOS box^34^. The analogies between the two neighboring genes extend in a way that their products, SSB and UvrA, both bind DNA and recruit other proteins onto it, which perform DNA repair. Finally, UvrA is also reported to suppress IR, acting in concert with RecQ^41^, analogously to SSB. The colocalization and coregulation of the *ssb* and *uvrA* genes are remarkable considering that *uvrA* shows neither with its partner *uvrB*, *uvrC,* and *uvrD* genes, indicating specially connected roles of the two genes in preserving genome stability, which is certainly worth elucidating further.

There are five human RecQ analogues, and the loss of function of any one of them causes severe illnesses such as Bloom, Werner, Rothmund-Thompson, etc., syndromes, which are characterized by gross genome instability, as reflected by increased cancer rates, premature aging, infertility, immunodeficiency, shortened lifespan etc. (reviewed in^42^). Thus, in addition to providing new insight into conserved mechanisms for genome preservation, our findings offer the possibility of new therapeutic approaches, such as varying/increasing the cellular level of a eukaryotic SSB analogue RPA to alleviate the requirement for RecQ activities, for treating cells with impaired RecQ function.

*E. coli* IR shares considerable similarity with the eukaryotic Microhomology-Mediated-End-Joining (MMEJ) pathway of DSB repair (discussed in^4^), which is mutagenic and a “major mechanism for chromosome translocations, and possible other rearrangements in mammalian cells”^43^. Such recurrent chromosome translocations are found in many malignancies^43^.

The common features of IR and MMEJ include their initiation by DSB resection (stemming from replication impairment)^10^. The ensuing 3’ overhangs then align broken DNA ends by an end-joining reaction dependent on microhomologies and ligase function^10,43^. Although both pathways are independent of a cognate recombinase (RecA/RAD51), they are actually suppressed by homology-dependent repair, and this competition is resolved during the DSB resection process^4,44^. Now we report another similarity between IR and MMEJ, namely, the suppression by their respective single-strand DNA binding proteins, SSB and RPA.

MMEJ suppression by RPA was shown to be caused by the inhibition of annealing between microhomologies^24,25^. However, the role of a yeast RecQ analog Sgs1 in RPA suppression of MMEJ was not analyzed, which is an interesting prospect since RPA is known to interact with Sgs1^45^ and many other cognate eukaryotic RecQ family members, e.g., hBLM^46^, WRN^47^ etc. Moreover, the RecQ core of the human BLM helicase managed to partially inhibit IR in the *E. coli* λ Spi^−^ assay^48^, indicating that aberrant DNA structures giving rise to IR fall within BLM helicase’s substrate range, which thus may be expected to disrupt (analogous) DNA intermediates resulting in MMEJ. Further elucidation is required concerning the role of eukaryotic RecQ family members in suppressing MMEJ, as well as their interaction with cognate RPA during this process.

## Materials and methods

### Strains, growth conditions and media

*E. coli* wild-type strain AB1157 and its derivatives (listed in Suppl Table S1) were grown in Luria–Bertani (LB) medium^49^ (supplemented with the appropriate antibiotics) at 30 °C until reaching the mid-logarithmical growth phase. The strains used in the λ Spi^−^ assay were lysogenic with a thermoinducible prophage λ*cI857*. The *ssb-1* allele codes for the mutant SSB-1 protein (His55 → Tyr), which is temperature sensitive^50^. SSB-1 gets rapidly inactivated by heating at 42 °C, but the reaction is reversible upon shifting the temperature below 30°C^51,52^. The likely cause of the  temperature sensitivity of the *ssb-1* mutant is the destabilization of SSB-1 tetramers with respect to monomers, hence their much lower affinity for ssDNA^36^.

### Construction of plasmids

The chromosomal *ssb* gene, including its natural promoters, was amplified by PCR from wild-type *E. coli* genomic DNA and cloned into the pACYC184 plasmid vector. The plasmids pID2 and pSID4 were constructed by cloning the insert into the Cam^30^ or Tc resistance genes, respectively. As depicted in Suppl Fig. S1, the pSID3 plasmid, expressing truncated SSB protein, was constructed by a PCR-based site-directed mutagenesis, using an unmodified forward primer (1) (5’-TAAAGTCGACGAGTGTTGTGTAACAATG-3’) upstream of the promoter region and a modified reverse primer (3) (5’- TAAAGGATCCTTAATCATCCACCTTAAAACAATATAACCTATTGTTTTAATGACAAATCACATCGGCGGC -3’) lacking the conserved 8 amino acid C-terminal tip sequence. The pSID1 was used as a negative control since it contains the intact *ssb* coding region, but lacks its promoter region. For this purpose, the forward primer (2) (5’-TAAAGTCGACATGGCCAGCAGAGGCGTA-3’) was designed downstream of the promoter region, and the reverse primer (4) (5’-TAAAGGATCCTTAATCATCCACCTTAAAAC-3’) targeted the terminal part of the *ssb* coding region. The sequence of the cloned fragments was checked by DNA sequencing.

### Transcription analysis

The bacteria were grown in LB medium (containing the appropriate antibiotics) at 30 °C with aeration until reaching OD_600_ ~ 0.4. The mRNA was isolated from the bacteria using Qiagen RNeasy Mini kit, according to the manufacturer’s protocol. RNA was quantified with the Quant-IT RNA assay kit using a Qubit fluorometer (Invitrogen, Waltham, MA, USA). RNA was quantified and converted into cDNA by reverse transcription (PrimeScript RT reagent Kit Takara, Dalian, China) using specific modified primers as described earlier^32^. The *ssb* and *sulA* expression in cells harboring the above-mentioned plasmids was determined by RT-qPCR, using primers (*ssb*-fw GTTGTGCTGTTCGGCAAACT and rev GCGATCCTGACCGCAATCAA, *sulA*-fw CCTGAACCCATTCGCCAGTG and rev GCCGGGCTTATCAGTGAAGT), and according to our improved protocol for transcriptome analysis, which does not rely on template DNA removal and is therefore more reliable and reproducible than the standard assay, especially in the case of prokaryotic genes and non-coding repetitive DNA in eukaryotes^32,53^. The following thermal cycling conditions were used: 50 °C 2 min; 95 °C 7 min; 95 °C 15 s; 60 °C 1 min for 40 cycles followed by dissociation stage: 95 °C for 15 s; 60 °C for 1 min; 95 °C for 15 s; and 60 °C for 15 s. Amplification specificity was confirmed by dissociation curve analysis and the specificity of amplified products was tested on agarose gel. Glyceraldehyde-3-phosphate dehydrogenase (GAPDH, ID:EG10367) was used as an endogenous control for normalization and was stably expressed without any variation among samples.

Amplifcation specificity was confrmed by dissociation curve analysis and the specifcity of amplifed products was tested using a control without a template. Post-run data were analysed using LinRegPCR sofware v.11.1. which enables calculation of the starting concentration of amplicon in the sample (“N0 value”). N0 value is expressed in arbitrary fuorescence units and is calculated by considering PCR efficiency and baseline fuorescence. The “N0 value” determined for each technical replicate was averaged, and the averaged “N0 values” were divided by the “N0 values” of the endogenous control.

### ***λ Spi***^***−***^*** assay***

A variation of the procedure developed by Ikeda et al.^8^ was used^4^. The bacteria were grown in LB medium supplemented with 10 mM MgSO_4_ to OD_600_ ~ 0.4 at 30 °C. Bacterial cultures were then incubated at 42 °C with aeration for 40 min to induce lytic cycle of λ*cI857* prophage and to inactivate the SSB-1 protein. Then, the bacteria were incubated at 37 °C with aeration for 120 min, until lysis occurred. Chloroform was added to the lysates, which were then centrifuged for 10 min at 10,000×*g*. The lysates were stored at 4 °C.

To determine the total phage titer, the lysates were serially diluted and incubated with AB1157 bacteria for 15 min at 42 °C. The bacteria were then mixed with soft trypticase agar, spread on trypticase plates and incubated overnight at 37 °C. The titer of λ Spi^−^ phage was determined by mixing lysates with the P2 lysogenic strain NM767 and incubated for 15 min at 42 °C, after which they were mixed with trypticase soft agar, spread on trypticase plates and incubated overnight at 37 °C. On each plate, either 2 or 3 × 10^8^ phages were added. For wt strain, on average, one large plaque appeared per 10 plates used (*i.e.,* one λ Spi^−^ phage per ~ 3 × 10^9^ phages). The frequency of λ Spi^−^ phage was determined by dividing the titer of λ Spi^−^ phage by the total phage titer.

### Transductional crosses

Inheritance of the chromosomal Pro^+^ marker was determined using P1 phages and a procedure modified with respect to the earlier one^54^. The *ssb-1* mutants were grown in LB medium at 30 °C until reaching OD_600_ ~ 0.3, when they were resuspended in MC buffer (100 mM MgSO_4_, 5 mM CaCl_2_) and infected with P1 at a multiplicity of 0.1 and incubated at 42 °C for 20 min. Afterwards, 5 mM Na-citrate was added, and incubation was prolonged for another 15 min at 42 °C. The mixtures were then spread on minimal M9 plates^49^ containing 5 mM Na-citrate and all of the required amino acids except proline. The plates were incubated at 42 °C for 60 min, and subsequently at 30 °C for 48 h. Control crosses were done at 30 °C for 30 min in MC buffer. Na-citrate was added and the mixtures were spread on M9 plates and incubated for 48 h at 30 °C. The relative HR frequency reduction was expressed as a ratio of the rate of Pro^+^ tranductants obtained in crosses at 30 °C to that at 42 °C.

## Supplementary Information

Supplementary Information.

## Data Availability

All data generated or analyzed during this study are included in this published article.
