# Supplementary material for: Single-strand DNA-binding protein suppresses illegitimate recombination in Escherichia coli, acting in synergy with RecQ helicase
Source: Sci Rep. 2024 Sep 3;14:20476. doi: 10.1038/s41598-024-70817-5 (PMC11372144; doi:10.1038/s41598-024-70817-5)
Supplement: Supplementary file 1 — Supplementary Information. [file 41598_2024_70817_MOESM1_ESM.docx]

**Supplemental Table 1** Bacterial strains used in this study.

| Strain | Relevant genotype | Reference or construction |
| --- | --- | --- |
|  | AB1157 and derivatives |  |
| AB1157 | Rec^+^, Pro^-^, F^-^ | 1 |
| DE110 | Δ*recQ*::*kan* | 2 |
| DE733 | *ssb-1* *malE145::*Tn*10* | P1.KL922 x AB1157 to Tc^r^, temp^s^ |
| DE735 | *ssb-1* *malE145::*Tn*10* Δ*recQ*::*kan* | P1.SWM1003 x DE733 to Km^r^ |
| DE658 | Δ*recD744*::FRT | Laboratory collection |
| DE734 | Δ*recD744*::FRT *ssb-1* *malE145::*Tn*10* | P1.KL922 x DE658 to Tc^r^, temp^s^ |
| DE749 | Δ*recD744*::FRT Δ*recQ*::*kan* | P1.SWM1003 x DE658 to Km^r^ |
| RIK174 | *recBD1080A* | 3 |
| DE150 | *recB1080* Δ*recQ*::*kan* | 2 |
| DE738 | *recB1080 ssb-1* *malE145::*Tn*10* | P1.KL922 x RIK174 to Tc^r^, temp^s^ |
| DE739 | *recB1080* Δ*recQ*::*kan ssb-1* *malE145::*Tn*10* | P1.KL922 x DE150 to Tc^r^, temp^s^ |
| LMM1728 | Δ(*recC-argA*)*234* Δ*sbcB*::*cam sbcD300*::*kan* | Davor Zahradka |
| DE760 | Δ(*recC-argA*)*234* Δ*sbcB*::*cam sbcD300*::*kan ssb-1* *malE145::*Tn*10* | P1.KL922 x LMM1728 to Tc^r^, temp^s^ |
| DE753 | Δ(*recC-argA*)*234* Δ*sbcB*::*cam sbcD300*::*kan recQ1803::*Tn*3* | P1.DE92 x LMM1728 to Ap^r^ |
| DE202 | *lexA3* Δ*malB::kan* | Laboratory collection |
| DE773 | *lexA3* Δ*malB::kan recQ1803::*Tn*3* | P1.DE92 x DE202 to Ap^r^ |
| DE105 | *rec^+^* λ*cI*857 | Lysogenization of AB1157 |
| DE111 | Δ*recQ*::*kan* λ*cI*857 | 2 |
| DE741 | *ssb-1* *malE145::*Tn*10* λ*cI*857 | Lysogenization of DE733 |
| DE743 | *ssb-1* *malE145::*Tn*10* Δ*recQ*::*kan* λ*cI*857 | Lysogenization of DE735 |
| DE754 | Δ*recD744*::FRT λ*cI*857 | Lysogenization of DE658 |
| DE742 | Δ*recD744*::FRT *ssb-1* *malE145::*Tn*10* λ*cI*857 | Lysogenization of DE734 |
| DE750 | Δ*recD744*::FRT Δ*recQ*::*kan* λ*cI*857 | Lysogenization of DE749 |
| DE775 | *lexA3* Δ*malB::kan* λ*cI*857 | Lysogenization of DE202 |
| DE776 | *lexA3* Δ*malB::kan recQ::*Tn*3* λ*cI*857 | Lysogenization of DE773 |
| DE153 | *recB1080* λ*cI*857 | Lysogenization of RIK174 |
| DE154 | *recB1080* Δ*recQ*::*kan* λ*cI*857 | 2 |
| DE746 | *recB1080 ssb-1* *malE145::*Tn*10* λ*cI*857 | Lysogenization of DE738 |
| DE762 | Δ(*recC-argA*)*234* Δ*sbcB*::*cam sbcD300*::*kan* λ*cI*857 | Lysogenization of LMM1728 |
| DE761 | Δ(*recC-argA*)*234* Δ*sbcB*::*cam sbcD300*::*kan ssb-1* *malE145::*Tn*10* λ*cI*857 | Lysogenization of DE760 |
| DE785 | Δ(*recC-argA*)*234* Δ*sbcB*::*cam sbcD300*::*kan recQ1803::*Tn*3* λ*cI*857 | Lysogenization of DE753 |
| DE105 | *rec^+^* λ*cI*857 pACYC184 | Tc^r^, Cm^r^ |
| DE105 | *rec^+^* λ*cI*857 pID2 (*ssb^+a^*/pACYC184) | Tc^r^ |
| DE105 | *rec^+^* λ*cI*857 pSID1 (-p, *ssb*/pACYC184) | Cm^r^ |
| DE105 | *rec^+^* λ*cI*857 pSID3 (*ssbΔC*/pACYC184) | Cm^r^ |
| DE105 | *rec^+^* λ*cI*857 pSID4 (*ssb^+^*/pACYC184) | Cm^r^ |
| DE111 | Δ*recQ*::*kan* λ*cI*857 pACYC184 | Tc^r^, Cm^r^ |
| DE111 | Δ*recQ*::*kan* λ*cI*857 pID2 (*ssb^+^*/pACYC184) | Tc^r^ |
| DE111 | Δ*recQ*::*kan* λ*cI*857 pSID1 (-p, *ssb*/pACYC184) | Cm^r^ |
| DE111 | Δ*recQ*::*kan* λ*cI*857 pSID3 (*ssbΔC*/pACYC184) | Cm^r^ |
| DE111 | Δ*recQ*::*kan* λ*cI*857 pSID4 (*ssb^+^*/pACYC184) | Cm^r^ |
| DE741 | *ssb-1* *malE145::*Tn*10* λ*cI*857 pSID1 (-p, *ssb*/pACYC184) | Cm^r^ |
| DE741 | *ssb-1* *malE145::*Tn*10* λ*cI*857 pSID3 (*ssbΔC*/pACYC184) | Cm^r^ |
| DE741 | *ssb-1* *malE145::*Tn*10* λ*cI*857 pSID4 (*ssb^+^*/pACYC184) | Cm^r^ |
| DE153 | *recB1080* λ*cI*857 pACYC184 | Tc^r^, Cm^r^ |
| DE153 | *recB1080* λ*cI*857 pID2 (*ssb^+^*/pACYC184) | Tc^r^ |
| DE154 | *recB1080* Δ*recQ*::*kan* λ*cI*857 pACYC184 | Tc^r^, Cm^r^ |
| DE154 | *recB1080* Δ*recQ*::*kan* λ*cI*857 pID2 (*ssb^+^*/pACYC184) | Tc^r^ |
| DE762 | Δ(*recC-argA*)*234* Δ*sbcB*::*cam sbcD300*::*kan* λ*cI*857 pACYC184 | Tc^r^, Cm^r^ |
| DE762 | Δ(*recC-argA*)*234* Δ*sbcB*::*cam sbcD300*::*kan* λ*cI*857 pACYC184 pID2 (*ssb^+^*/pACYC184) | Tc^r^ |
| DE785 | Δ(*recC-argA*)*234* Δ*sbcB*::*cam sbcD300*::*kan recQ::*Tn*3* λ*cI*857 (pACYC184) | Tc^r^, Cm^r^ |
| DE785 | Δ(*recC-argA*)*234* Δ*sbcB*::*cam sbcD300*::*kan recQ::*Tn*3* λ*cI*857 pID2 (*ssb^+^*/pACYC184) | Tc^r^ |
| DE775 | *lexA3* Δ*malB::kan* λ*cI*857 pSID1 (-p, *ssb*/pACYC184) | Cm^r^ |
| DE775 | *lexA3* Δ*malB::kan* λ*cI*857 pSID3 (*ssbΔC*/pACYC184) | Cm^r^ |
| DE776 | *lexA3* Δ*malB::kan recQ::*Tn*3* λ*cI*857 pSID1 (-p, *ssb*/pACYC184) | Cm^r^ |
| DE776 | *lexA3* Δ*malB::kan recQ::*Tn*3* λ*cI*857 pSID3 (*ssbΔC*/pACYC184) | Cm^r^ |
| DE776 | *lexA3* Δ*malB::kan recQ::*Tn*3* λ*cI*857 pSID4 (*ssb^+^*/pACYC184) | Cm^r^ |
|  | Other strains |  |
| KL922 | *ssb-1* (ts) *malE145::*Tn*10* | Laboratory collection |
| SWM1003 | Δ*recQ*::*kan* | 4 |
| NM767 | P2 lysogen | Noreen E. Murray |

^a^ Plasmid genotype designations *ssb*^+^; -p, *ssb*; and *ssb*ΔC represent: promoters with whole coding region; coding region without promoters and promoters with truncated coding region, respectively.

**Supplemental** **Figure 1**


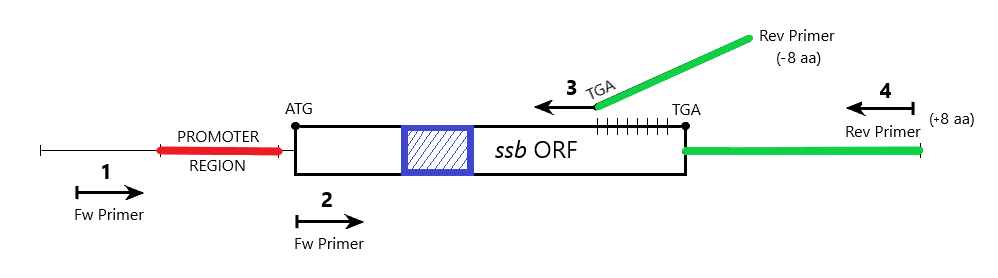


**Figure 9** Primers used for the construction of *ssb* overproducing plasmids: pSID1 (primers 2 and 4), pSID3 (primers 1 and 3) and pSID4 (primers 1 and 4). Blue box represents *ssb* gene region used for transcription quantification by RT-qPCR.

**REFERENCES**

1. Bachmann, B.J. (1972). Pedigrees of some mutant strains of *Escherichia coli* K-12. *Bacteriol*. *Rev*. **36**, 525–557.
2. Ivanković, S., Đermić, D. (2012). DNA end resection controls the balance between homologous and illegitimate recombination in *Escherichia coli*. *PLoS One*. **7**, e39030.
3. Jockovich, M.E., Myers, R.S. (2001). Nuclease activity is essential for RecBCD recombination in *Escherichia coli*. *Mol. Microbiol.* **41**, 949-962.
4. Mendonca, V.M., Klepin, H.D., Matson, S.W. (1995). DNA helicases in recombination and repair: construction of a Δ*uvrD* Δ*helD* Δ*recQ* mutant deficient in recombination and repair. *J. Bacteriol*. **177**, 1326-1335.
